# Extracellular Vesicles in Sport Horses: Potential Biomarkers and Modulators of Exercise Adaptation and Therapeutics

**DOI:** 10.3390/ijms26094359

**Published:** 2025-05-03

**Authors:** Dominika Milczek-Haduch, Magdalena Żmigrodzka, Olga Witkowska-Piłaszewicz

**Affiliations:** 1 Department of Large Animals Diseases and Clinic, Institute of Veterinary Medicine, Warsaw University of Life Sciences, Nowoursynowska 166, 02-787 Warsaw, Poland; 2 Department of Morphological Sciences, Institute of Veterinary Medicine, Warsaw University of Life Sciences, Nowoursynowska 166, 02-787 Warsaw, Poland; 3 Department of Pathology and Veterinary Diagnostic, Institute of Veterinary Medicine, Warsaw University of Life Sciences, Nowoursynowska 166, 02-787 Warsaw, Poland; magdalena_zmigrodzka@sggw.edu.pl

**Keywords:** EVs, exercise physiology, muscle adaptation, recovery, nanostructures

## Abstract

Significant systemic metabolic benefits result from even a single exercise session by activating multiple metabolic and signaling pathways within the organism. Among these mechanisms, extracellular vesicles (EVs) play a critical role by delivering their molecular cargo to neighboring or distant cells, thereby influencing cellular metabolism and function. As research progresses, EVs represent an exciting frontier in exercise science and fitness adaptation processes. There is increasing interest in understanding the physiology of EVs as signaling particles and their use as minimally invasive diagnostic and prognostic biomarkers in the early detection of oxidative stress-related abnormalities. They also show potential to be used in monitoring exercise progress, injury prevention, or recovery, and may provide insights for personalized training programs. This review examines the current understanding of the role of physical activity in generating exercise-responsive EVs. It highlights the potential applications of EVs in exercise science and personalized fitness optimization, not only for human athletes but also for exercising animals such as horses. On the other hand, it also presents potential difficulties that researchers currently working on this topic may encounter due to technical limitations.

## 1. Introduction

For many years, studies have explored the impact of physical activity on the human body. Regular, moderate physical activity, when appropriately tailored to an individual’s age and fitness level, is well known to have multifaceted health benefits [1]. Exercise initiates a cascade of physiological responses, initially disrupting homeostasis and triggering stress responses and signaling pathways that drive various adaptations [2]. Over time, these adaptations enhance fitness, improving the efficiency and effectiveness of physical performance [3]. These adaptive changes span multiple levels, including cellular, cardiovascular, respiratory, muscular, and metabolic systems [2,4].

Skeletal muscles, comprising up to 40% of body weight in most mammals, function as an endocrine organ, releasing muscle-derived factors called myokines that help regulate the body’s response to physical activity. Two main mechanisms mediate these systemic benefits: cytokine release from contracting muscles [5,6], a classic pathway targeting the endoplasmic reticulum [7], and an additional pathway involving extracellular vesicles (EVs) [8]. This novel EV-mediated communication system may work alongside myokines to transmit exercise’s benefits to other tissues, although studies on this form of intercellular communication are still limited.

EVs create a complex intercellular communication network that surpasses traditional signaling pathways, enabling nuanced, targeted exchanges of information and cargo [9]. The lipid bilayer of EVs protects their bioactive molecules from enzymatic degradation, allowing intact delivery to recipient cells [10]. Traveling through blood, lymph, and cerebrospinal fluid, EVs can reach distant cells and tissues, facilitating communication across non-connected cells [11]. Carrying diverse bioactive molecules, EVs participate in numerous biological processes, influencing recipient cell behavior in various ways.

The importance of EV-mediated communication is further evidenced by their increased production in diseases like cancer, neurodegenerative disorders, cardiovascular diseases, and inflammation [12,13]. Understanding and manipulating EV interactions could open new avenues for therapies. By transferring nucleic acids like microRNAs, EVs can alter gene expression and cellular behavior, with implications for cellular reprogramming, tissue regeneration, and disease modulation [14]. EVs, isolatable from various body fluids, hold potential as non-invasive diagnostic tools, offering insight into cellular and tissue states. Moreover, they show promise in drug delivery, with possibilities for engineering EVs to carry specific cargo to target cell types [15].

This review thus examines the effects of physical activity on EV generation in humans and animals, their potential roles, and future research directions.

## 2. Biological Basis of EVs

### 2.1. Biogenesis, Release, and Classification of EVs

**Figure 1:**
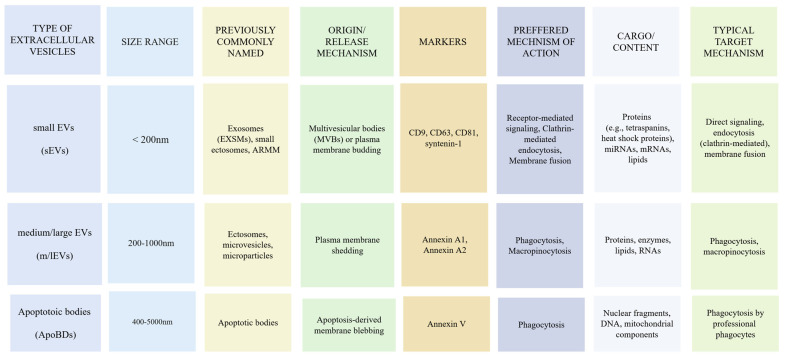
Classification of EVs.

EVs are cell-derived particles with a lipid bilayer, lacking a nucleus. They can be classified by cellular origin, physical characteristics, or biochemical composition, with terms like microvesicles (MVs), microparticles (MPs), ectosomes, oncosomes, and exosomes (EXSMs) frequently used [16,17]. According to the International Society for Extracellular Vesicles (ISEV) guidelines (Minimal Information For Studies Of Extracellular Vesicles 2018 and 2023—MISEV2018 and MISEV2023), terms like EXSMs and ectosomes should only be used if subcellular origin is confirmed [18]. Otherwise, classification by EV size is preferred, with small EVs (sEVs) under 200 nm and medium/large EVs (m/lEVs) over 200 nm, with the isolation method specified (Figure 1) [18,19,20].

EV release is often triggered by cell activation, injury, inflammation, or stress [21]. The biogenesis of sEVs and m/lEVs differs but is consistent across cell types. m/lEVs, previously known as ectosomes or microvesicles, form by outward budding from the cell’s plasma membrane. This process, driven by actin and myosin rearrangement, involves the bulging and pinching of the plasma membrane through the action of proteins like dynamin to create spherical microvesicles [22,23,24]. Once released, m/lEVs interact with other cells through body fluids, acting in autocrine or paracrine communication and carrying bioactive molecules such as mRNAs, miRNAs, proteins, and enzymes [25].

sEVs (also frequently named EXSMs) are not limited to exosomes but typically include small ectosomes (30–150 nm), ARMMs (Arrestin domain-containing protein 1-mediated microvesicles), and other vesicles smaller than 200 nm, with different mechanisms of biogenesis [25,26,27]. Initially, membrane-bound molecules are enclosed within an early endosome, which matures into and becomes a late endosome or multivesicular body (MVB).

The membrane of the MVB invaginates, creating even smaller vesicles called intraluminal vesicles (ILVs) inside the MVB’s interior. This process is primarily mediated by the endosomal sorting complex required for transport (ESCRT) machinery and associated proteins such as ALIX and syntenin, which facilitate membrane budding and scission [28,29,30]. The MVB eventually fuses with the plasma membrane, releasing ILVs (now termed EXSMs) into the extracellular space, thus enabling communication with distant cells. The release of EXSMs is a dynamic and regulated process that can be influenced by various factors, including cell type, cellular state, and external stimulation like ectosome formation [26,27]. In previous studies, older EV terminology focused mostly on smaller EVs based on size rather than confirmed biogenesis. However, in this review, we are going to use the newest classification, and when the subcellular origin could not be proven, we decided to use EVs in general.

Apoptotic bodies (ApoBDs) are larger EVs (500–4000 nm), released only during apoptosis. As cells undergo apoptosis, chromatin condenses, membranes bleb, and cellular components form membrane-bound vesicles called apoptosomes, some of which contain DNA that can transfer to recipient cells after phagocytosis. While sEVs and m/lEVs are secreted during active processes in the cell, apoptotic bodies form only during programmed cell death [28,29].

The cargo within EVs is diverse and selective, reflecting the originating cell’s status and activities and including proteins, nucleic acids (RNA and DNA), and lipids [30]. EV proteins cover a wide array of biomolecules with varied functions. Biogenesis and sorting proteins facilitate cargo formation within EVs, with the ESCRT and proteins like Alix and TSG101 playing roles in ILV formation within endosomes [31]. Membrane and structural proteins in EXSMs maintain EV structure, with tetraspanins (CD9, CD63, CD81), integrins, and annexins aiding in cell adhesion and membrane fusion [32]. Proteins involved in signaling pathways, such as growth factors, receptors, and ligands [33], can activate or modulate cascades in recipient cells upon EV uptake. Enzymatic proteins (proteases, lipases, kinases) catalyze reactions in recipient cells, influencing cellular processes upon EXSM internalization [34]. Heat shock proteins (HSP90, HSP70) support protein folding and stability under stress, aiding donor cell protection [35]. EVs with MHC I/II and immune checkpoint molecules (e.g., PD-L1) can also modulate immune responses in recipient cells [36,37].

Lipids are crucial for EVs’ structure and function, with their composition influenced by cell type, physiological conditions, and the microenvironment. Phospholipids form the lipid bilayer, protecting cargo and allowing recipient cell interactions, while sphingolipids (SM, ceramides, glycosphingolipids) add structural integrity. Cholesterol, embedded in the membrane, regulates phospholipid packing, impacting EVs’ fluidity, stability, and shape [38,39]. Nucleic acids, including RNA and DNA, contribute to various cellular functions. Although EVs contain mRNA and rRNA, miRNA significantly impacts target cells [40]. EVs’ DNA content, though less studied than RNA, is an emerging research area [41]. Thus, EV cargo shares some similarities across sizes but shows notable differences based on unique biogenesis and release mechanisms.

In summary, EVs are diverse nanoscale vesicles with biologically active cargo, released by cells into the extracellular space. According to MISEV18 and MISEV2023 guidelines, EXSMs, microvesicles, and ApoBDs should only be classified by subcellular origin if confirmed; otherwise, they should be classified by size.

### 2.2. Mechanisms of Extracellular Vesicle-Mediated Cargo Delivery and Cellular Modulation

EVs employ multiple mechanisms to influence recipient cells by transferring bioactive cargo and facilitating intercellular communication. The first mechanism is receptor-mediated signaling via ligands on EVs (glycoproteins, integrins, immunoglobulins, and lectins) that interact with specific transmembrane receptors on the recipient cell. This interaction has high specificity, causing a conformational change in the receptor, which activates signaling cascades inside the recipient cell [42]. These cascades involve downstream molecules like kinases and phosphatases, transmitting signals to affect gene expression, protein activity, and cellular responses, including metabolism, proliferation, differentiation, or apoptosis, depending on the pathways involved [43,44]. This mechanism is particularly common for small EVs like exosomes, which are enriched in membrane-associated ligands [25].

The second mechanism involves EV fusion with the recipient cell membrane. EVs use high-affinity surface proteins, such as MHC I and fusogenic proteins (syncytin-1 and syncytin-2), that undergo conformational changes to bring membranes into close proximity. This leads to the formation of a hemifusion pore, allowing lipid intermingling at the site of fusion. Subsequent rearrangements result in complete membrane fusion, releasing EV cargo directly into the recipient cell cytoplasm, thereby influencing cellular processes and functions [45,46]. Fusion is more likely for vesicles with appropriate fusogenic machinery, often seen in exosomes derived from multivesicular endosomes.

The third mechanism is internalization via endocytosis, including clathrin-dependent pinocytosis and phagocytosis. Specialized cells like macrophages preferentially use phagocytosis to internalize larger EVs, while pinocytosis is a more universal process for smaller vesicles. Upon internalization, the fate of EVs varies—they may fuse with endosomes and release their cargo into the cytoplasm or be targeted for degradation in lysosomes. It is worth noting that different types of EVs may prefer different routes: for example, larger ectosomes (150–1000 nm) are often internalized by phagocytosis, while smaller exosomes (<150 nm) are frequently taken up via clathrin-mediated endocytosis [25]. In both processes, the EVs are engulfed by the recipient cell membrane, forming vesicles that either release their cargo into the cytoplasm or direct them to lysosomes for degradation. Once internalized, EV cargo (microRNAs, mRNAs, proteins, lipids) can modulate gene expression, signaling pathways, and other cellular behaviors within the recipient cell [30].

Overall, the preferred mechanism of interaction depends on the EV subtype, its size, surface composition, and the type of recipient cell involved (Figure 1).

### 2.3. Time Course of Circulating EV Release in Relation to Exercise Type

**Figure 2:**
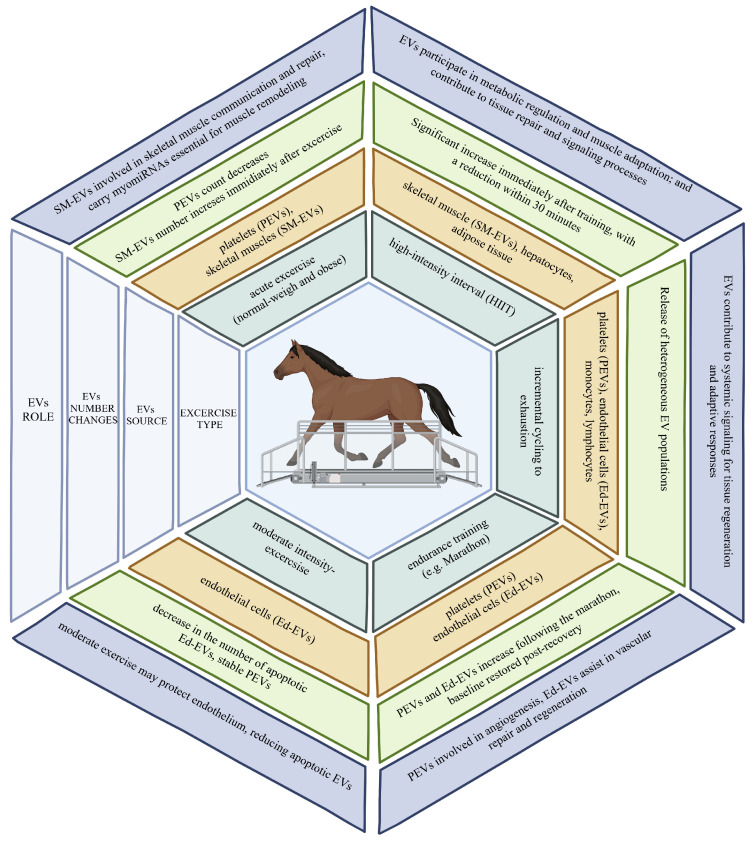
EV count variations based on exercise type.

EVs carrying muscle-specific miRNA, known as myomiRNA or myomiRs, are present in human plasma [47,48]. In sports medicine, the dynamics of EV numbers in response to exercise intensity, quality, and type are particularly relevant (Figure 2). However, our understanding of how exercise type influences EV release timing remains limited. Platelet-derived EVs (PEVs) in plasma rise during exercise, peaking within minutes to two hours post-exercise before returning to baseline [49,50]. In contrast, endothelial-derived EVs (EdEVs) show variable responses; some studies report an increase, peaking between 45 and 90 min post-exercise, while others do not [51,52].

Notably, high-intensity, long-duration exercise may increase EdEVs (CD31+ ANX5+ CD41-), potentially indicating vascular damage, while moderate exercise decreases ApoBDs from endothelial cells in healthy women, suggesting endothelial protection at lower intensities [53,54]. Frühbeis et al. observed that small EVs (sEVs, or EXSMs, 100–130 nm) rose immediately after cycling but normalized within 90 min. Treadmill running induced a more sustained increase in sEVs, with lactate measurements indicating that aerobic exercise prompts sEV release [55]. High-intensity interval training (HIIT) significantly increases skeletal muscle-, liver-, and adipose-derived EVs post-training, with numbers decreasing within 30 min, implying either uptake by target cells or degradation [56].

In post-exercise samples from normal-weight and obese subjects, PEV counts were lower, and skeletal muscle-derived EVs (SM-EVs) were higher, independent of gender [57]. Metabolic conditions also affect EV release, with a diverse EV population observed following exhaustive cycling, including PEVs, EdEVs, and immune cell-derived EVs [58]. Researchers propose that these variable EVs participate in multi-system signaling, supporting regeneration and adaptive responses. Further research is essential to explore additional influences beyond exercise type, duration, and intensity, as well as gender and training level, on EV quantity and composition. Comparative changes in the number of circulating EVs depending on the type and intensity of physical exercise are presented in Figure 2.

## 3. Role of EVs in Athletic Performance Enhancement

Optimal athletic performance relies on the efficient function of multiple organ systems, including the vascular system. Physical activity influences various hematological parameters, including blood cell counts, coagulation, and EV quantity and quality. As discussed earlier, changes in EV numbers are linked to the type and intensity of exercise, suggesting their role as modulators in cell communication, potentially impacting health and performance. Table 1 summarizes how EVs transport specific molecular markers and highlights the potential use of EVs as non-invasive biomarkers for monitoring fitness progress, oxidative stress, and tissue injury, offering a promising tool for personalized training and injury prevention.

Skeletal muscle (SM) constitutes around 40% of body weight, increasing to 53–57% in athletic animals like Greyhounds and Thoroughbreds [59,60]. During exercise, SM acts as a secretory organ, producing myokines that impact the body’s adaptation to physical activity through autocrine, paracrine, or endocrine pathways. EVs carry newly identified myokines previously undetectable in human plasma [61,62,63]. SM tissue in vitro secretes Myo-miRNAs (miR-1, miR-133a/b, and miR-206) encapsulated in EVs, with release levels varying by muscle type [64]. Downregulation of miR-133a/b in one muscle group reduces levels in neighboring muscles, supporting paracrine SM-EV activity.

EV-associated miRNAs, such as miR-206, miR-133b, and miR-146a, increase with exercise intensity and appear linked to SM remodeling rather than tissue damage [64,65,66]. Present in the skeletal muscle interstitium, SM-EVs are abundant and have miRNAs that suppress the expression of crucial in myogenesis PAX7 transcription factor [65]. Additionally, miRNA expression varies with training level, as shown in well-trained versus sedentary older men following exercise [67]. EVs present in fetal bovine serum (FBS) can influence proliferation and differentiation in SM cells in vitro, and EV-depleted FBS induced myostatin expression in C2C12 myoblasts, weakening differentiation and myotube formation [68]. This suggests EVs transfer regulatory molecules between species, potentially reflecting evolutionarily conserved mechanisms.

**Table 1:** EVs as molecular cargo transporters in exercise.

EV Source	Molecular Cargo	Role in Exercise Adaptation	Potential Use as Biomarkers	Reference
Skeletal Muscle EVs (SkM-EVs)	miRNAs (miR-1, miR-133a, miR-206), proteins	These miRNAs regulate muscle adaptation, myogenesis, and tissue repair during and after exercise	Can indicate muscle damage, repair, and remodeling post-exercise	[66,69]
Platelet EVs (PEVs)	VEGF, HGF, PDGF	Supports angiogenesis and enhances vascular response to exercise, helping oxygen and nutrient delivery	Can be used to monitor recovery, vascular health, and response to endurance training	[70,71]
Endothelial EVs (Ed-EVs)	MMP-2, MMP-9, miR-126	Promotes vascular remodeling, essential for sustaining increased blood flow during prolonged exercise	Useful in detecting vascular health and adaptation to endurance exercises	[72,73]
Exercise-Derived EVs (Ex-EVs)	Proteins (PI3K, ERK), miRNAs, lipids	Mediates systemic metabolic adaptations and intercellular communication for energy utilization and tissue repair	Potential non-invasive biomarkers for fitness level, oxidative stress, and early detection of overtraining	[53,58,74]
Adipose Tissue EVs	Exerkines, miRNAs	Plays a role in fat metabolism and energy homeostasis during exercise	Can be used to monitor metabolic adaptations to exercise	[75,76]

### 3.1. Antioxidant Potential of EVs

Skeletal muscle (SM) contractions increase oxidative stress due to higher production of reactive oxygen species (ROS) and free radicals (FRs). Studies show that even short, high-intensity muscle activity raises ROS in SM and blood, regulating fatigue, and forces generation in response to training [69]. ROS and FR production during exercise can have both positive and negative physiological effects. Evidence suggests that EV secretion and cargo content are influenced by oxidative stress, affecting redox status in recipient cells [77]. For instance, EVs have reduced levels of catalase (CAT), SOD2 (superoxide dismutase 2), and heat shock factor 1 (HSF1) after short aerobic training compared to untrained controls [78]. Additionally, blood polymorphonuclear cells from trained individuals have a higher pro-oxidant status, and their EVs carry antioxidant enzymes like CAT and PRDX2 following HIIT [56,79].

Exercise-derived EVs (Ex-EVs) also protect ECM components like collagen and hyaluronan from ROS damage, crucial for tissue integrity. In diabetes models, EVs with high SOD levels promote angiogenesis by influencing redox-sensitive pathways in endothelial cells, suggesting that EVs can indicate tissue resilience and vascular adaptation to exercise [80]. Neutrophil-derived EVs are rich in extracellular SOD, protecting matrix components like collagen and hyaluronan from oxidative damage [81]. EVs with increased SOD levels also promote angiogenesis, as seen in exercise-treated human and mouse plasma in a diabetes model [82]. Antioxidants in EVs, such as SOD and glutathione, are key to managing ROS, muscular fatigue, and cellular senescence post-exercise [83].

Redox-sensitive microRNAs (miRs), especially muscle-derived ones (e.g., miR-133, miR-206), respond to oxidative stress, aiding in muscle adaptation, repair, and anti-inflammatory response [84]. Monitoring miRNA changes in EVs could provide insights into the impacts of varying exercise intensities.

Exercise increases the NAD+/NADH ratio, which activates sirtuin (SIRT1), linked to longevity and mitochondrial function. EVs carry extracellular NAMPT (eNAMPT), which boosts NAD+ synthesis in recipient cells, supporting cellular adaptation [85]. Elevated eNAMPT levels in EVs may reflect an individual’s fitness level and training adaptation.

In summary, EVs provide a non-invasive means to monitor exercise-induced chain redox-sensitive molecules like antioxidant enzymes, miRNAs, and NAD+-related factors that reflect cellular adaptations. This insight could help design personalized training programs and minimize oxidative stress-related risks.

### 3.2. EVs as Metabolic Regulators

Endurance training enhances insulin responsiveness in skeletal muscles (SMs) over time. This increase in glucose uptake and metabolism is due to repeated activity, which boosts the expression and activation of key signaling molecules. EVs play an endocrine-like role by transporting biologically active molecules (exerkines). Among the 35 most common exerkines, 51% are listed on www.exocarta.org and 80% on www.microvesicles.org, underscoring the metabolic regulatory potential of EVs [75]. Exercise-induced EV cargo changes have been shown to prevent the transmission of insulin resistance [76]. Additionally, acute exercise reduces EV counts (PEVs, EdEVs, leukocyte-derived EVs, and tetraspanin EVs), while insulin infusion further decreases PEVs and EdEVs post-exercise, suggesting exercise lowers cardiovascular risk through insulin-mediated EV changes [74].

Aging affects metabolic homeostasis, decreasing NAD+ production and reducing extracellular nicotinamide phosphoribosyltransferase (eNAMPT) levels. EV-eNAMPT levels correlate with physical fitness and decline with age. In fit individuals, EV-eNAMPT can increase NAD+ and SIRT1 activity in vitro in C2C12 myoblasts, showing EVs’ role in maintaining metabolic balance [85].

### 3.3. Angiogenic Potential of EVs

Cardiovascular endurance training is a strong stimulus for angiogenesis, increasing capillary density in skeletal muscles to enhance blood flow and oxygen delivery during exercise [86]. This physiological process, essential for growth and wound healing, is regulated by a balance of pro- and antiangiogenic factors. Vascular endothelial growth factor (VEGF) signaling plays a key role, along with other factors like PDGF (platelet-derived growth factor), FGF (fibroblast growth factor), and EGF (endothelial growth factor), which support endothelial proliferation and migration in angiogenesis [87]. Under hypoxic conditions or during high-load resistance exercise (HLRE), EVs are released, promoting vasculogenesis [72].

Exercise-derived EVs (Ex-EVs) have been shown to enhance endothelial cell proliferation, migration, and tube formation in vitro. PEVs, the most abundant Ex-EV type, carry proangiogenic factors such as PDGF, VEGF, and FGF-2, which have both therapeutic (e.g., in myocardial ischemia) and potentially harmful effects, such as supporting angiogenesis in cancers [17,50,88]. While VEGF was not found in skeletal muscle-derived EVs (SkM-EVs), PEVs upregulate VEGF and other factors in mouse lung cancer models, indicating different EV roles in angiogenesis [89,90]. In the resting muscles, VEGF level is controlled by the estrogen-related receptor γ (ERRγ), whereas upon exercise, it is controlled by peroxisome proliferator-activated receptor gamma co-activator 1-alpha (PGC-1a), which is moderated by aerobic activity. Energy deficit caused by exercise activity produces and activates P/AMP-activated kinase which phosphorylates PGC-1a [70,91]. Moreover, when the NAD+/NADH ratio increases, it results in PGC-1a deacetylation and increased sirtuin activity [92]. Additionally, it was confirmed that Ed-EVs transfer MMP-2 (matrix metalloproteinase), MMP-9, and integrin 1, inducing endothelial cell adhesion and tube-like structure formation in vitro [93]. It proved that Ed-EVs act as auto- and paracrine mediators. Moreover, under the influence of pro-inflammatory IL-3 (Interleukine), ECs release Ed-EVs with miR-126-3p and Stat5 protein. These proangiogenic miRNAs are then horizontally transferred to vascular smooth muscle cells to exert angiogenic properties [71].

Endurance training increases intraluminal shear stress (ISS), stimulating nitric oxide-induced vascular dilation. High ISS decreases Ed-EVs, while low ISS increases their number. PEVs levels, however, increase under both conditions. Marathon running raises both PEV and Ed-EV numbers compared to basal levels in healthy individuals, highlighting physical activity’s vascular impacts [94].

Conversely, just 5 days of reduced physical activity in active individuals significantly increased apoptotic Ed-EVs, suggesting a link between inactivity and endothelial dysfunction [95]. Changes in miRNA expression, particularly those regulating VEGF and glutathione metabolism, also contribute to angiogenesis. Exercise-induced increases in miRNAs within EVs, as well as upregulation of angiopoietin-1 (ANG1) and its receptor TIE2, initiate a proangiogenic pathway, enhancing adaptation in skeletal muscle [73,96].

As shown in Table 2, the transport of proangiogenic factors like VEGF, HGF, and PDGF by EVs and their role in facilitating new blood vessel formation and muscle repair post-exercise make EVs crucial in recovery and performance optimization.

**Table 2:** Role of EVs in angiogenesis and muscle regeneration.

EV Source	Key Factors Transported by EVs	Role in Angiogenesis	Role in Muscle Regeneration	References
Platelets (PEVs)	VEGF, HGF, PDGF, FGF-2	Facilitates new blood vessel formation (angiogenesis) by promoting endothelial cell proliferation and migration	Enhances tissue regeneration by improving oxygen and nutrient supply to muscles	[50,87,88,89,97,98,99]
Endothelial Cells (Ed-EVs)	MMP-2, MMP-9, Integrins	Promotes endothelial cell adhesion and tubule formation, vital for new capillary networks	Supports tissue repair by increasing vascularization in damaged areas	[71,93,97,98]
Skeletal Muscle EVs (SkM-EVs)	miR-206, miR-133b, miR-146a	Does not promote angiogenesis directly, but involved in muscle communication and repair	Delivers myomiRNAs to support muscle cell proliferation and differentiation, crucial for muscle repair	[89,90,97,100,101]
Exercise-Derived EVs (Ex-EVs)	PI3k, ERK, VEGF, ANG1, TIE2	Enhances endothelial proliferation and migration, leading to better blood flow and oxygen supply to exercising muscles	Plays a significant role in long-term muscle adaptation and injury recovery	[72,73,96,97,98]

### 3.4. Immunomodulatory Role of Exercise-Released EVs

**Figure 3:**
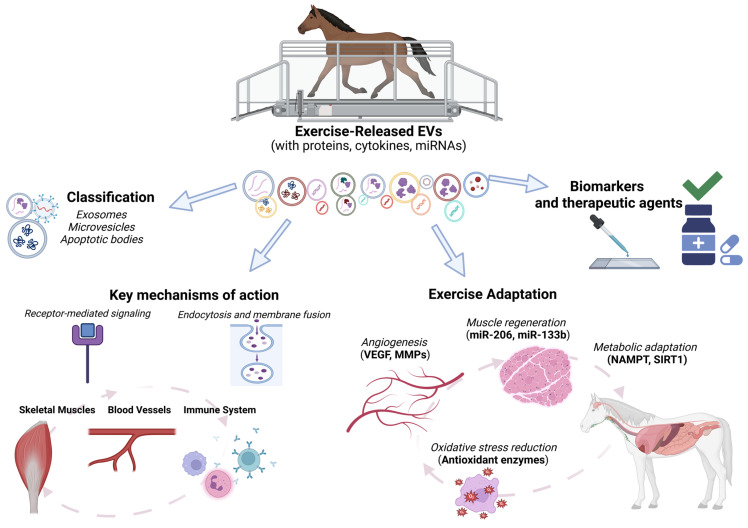
Immunomodulatory effects of exercise-induced EVs.

Immune modulation adjusts immune activity to maintain homeostasis in response to internal and external factors. EVs are known to influence immune responses by exerting both pro- and anti-inflammatory effects (Figure 3). Exercise stimulates the release of EVs from skeletal muscle cells containing signaling molecules like miRNAs and cytokines/myokines with anti-inflammatory properties. These EVs, released into the extracellular matrix, can act paracrinally or travel via blood to distant cells, helping reduce inflammation in the host.

Studies show that exercise significantly affects the expression of chemokines and interleukins in EVs, including those related to inflammation (CCL17, CXCL1, IL-18, IL-1RA), angiogenesis (ANG1, TIE2), and hemostasis (PAR-1, SELP, SRC) [96]. Exercise alters 54 proteins in plasma EVs, while the EV-free plasma proteome shows only four changes. Anti-inflammatory cytokines like IL-10 and IL-6 with broad effects are also transported in EVs during exercise, and meteorin-like proteins in EVs inhibit pro-inflammatory cytokines such as IL-1β and IL-18 [102,103].

Meanwhile, Catitti et al. confirmed that SkM-EVs contain mRNAs involved in inflammatory responses [102]. SkM-EVs induce miRNA expression of pro-inflammatory IL-8 and monocyte chemoattractant protein 1 (Mcp1). Higher EV production was noted in oxidative muscles, which also showed greater angiogenesis compared to glycolytic muscles [88].

## 4. EVs as Biomarkers for Monitoring and Assessing Fitness Adaptation

EVs are gaining attention as potential biomarkers across various medical fields, including for diagnosing and monitoring cancer, neurodegenerative, and cardiovascular diseases [104,105]. While promising, using EVs to monitor and assess fitness adaptation is still developing. Further research is required to standardize methods, identify specific EV markers, and validate their clinical relevance, positioning EVs as a non-invasive tool for tracking physiological responses to exercise. EVs carrying specific proteins or nucleic acids can signal muscle damage and repair processes.

One study examined redox homeostasis in exercise adaptation via EVs as communication pathways, analyzing total EV number, size, and cargo redox content (e.g., antioxidants, transcription factors, HSPs). Findings suggest aerobic capacity influences EV redox status and that short-term aerobic training modifies it, indicating potential for EVs as performance markers and tools for early detection of oxidative stress during training [78].

Although the potential of EVs as biomarkers in exercise adaptation is promising, it is important to note that many variables influence the results of the studies. EV cargo, particularly in exosomes and muscle-derived EVs, shows gender-specific variations in response to resistance exercise [106]. Additionally, studies under simulated military stress identified dimorphic EV profiles in men and women [107]. Despite these variables, trends are emerging to define exercise-related biomarkers. For instance, treadmill studies on rats showed increased sEVs and altered sRNA profiles post-exercise, highlighting vesicular sRNAs’ potential role in adaptation [108]. Changes in EV quantity, size, and cargo during acute training could also aid in identifying markers for overtraining syndrome (OTS), a condition still lacking definitive biomarkers in human and equine sports [109,110]. The relationship between gene expression in plasma EVs may reveal indicators for training performance and safety, enabling personalized exercise interventions. Blood EV content changes could help non-invasively assess muscle injury after exercise.

In summary, EVs hold great promise as biomarkers for assessing fitness adaptation, injury, and overtraining. Their role extends beyond exercise physiology, as they have shown potential in diagnosing and monitoring various diseases, including cancer, neurodegenerative, and cardiovascular conditions [111]. Delineating the role of EVs in each of these areas is advisable to highlight their broader physiological importance. In cancer, EVs can carry tumor-specific molecules, aiding in early diagnosis and the monitoring of treatment response. In neurodegenerative diseases, they can reflect neural cell health and progression, while in cardiovascular diseases, EVs are involved in endothelial function and inflammatory response. Together, these insights underscore EVs’ unique capacity to serve as non-invasive biomarkers across multiple systems, demonstrating their vital role in health and disease.

## 5. Therapeutic Potential of EVs in Sports Medicine

In response to exercise-induced injuries, circulating EVs undergo alterations in their miRNA content, marking muscle damage and potentially serving as biomarkers for conditions like Duchenne muscular dystrophy and myotonic dystrophy [97,100,101]. The cyclic muscle damage regeneration process observed in these myopathies suggests that monitoring EV-derived miRNAs could offer an objective method for tracking disease progression [97]. Skeletal muscle regeneration following repetitive microinjury is a complex yet crucial physiological process in which EVs, particularly those carrying muscle-specific miRNAs (myo-miRNAs), play a critical role. Satellite muscle cells (SMSCs), located beneath the basal lamina, work with EVs to reduce inflammation and promote repair by activating, proliferating, and differentiating other muscle cells [97,98].

The bilayer structure of EVs protects sensitive free-circulating miRNAs from RNase degradation, making EVs a pivotal component in regenerative medicine. Although selective loading mechanisms of myo-miRNAs into EVs remain poorly understood, they are crucial for advancing muscle regeneration research [98]. SMSC-derived EVs, in particular, stimulate extracellular matrix genes like MMP-9, thereby supporting muscle growth and regeneration [99,112]. For example, EVs secreted during 30 Hz electrically stimulated tetanic contractions in C2C12 mouse myotubes showed increased levels of ALG-2 interacting protein X (Alix), a marker associated with EV secretion and muscle regeneration [113].

Beyond promoting muscle cell proliferation, EVs support broader tissue regeneration, essential for muscle functionality beyond the number of myocytes. This function is significant in conditions like tendinopathies, where slow healing rates often fail to restore the pre-injury range of motion. The development of EV-based treatments for ligaments and tendons could enhance non-operative healing or improve surgical outcomes. EXSMs, for instance, play a role in modulating inflammation, promoting macrophage polarization, and regulating gene expression, thereby remodeling the extracellular environment and promoting angiogenesis—processes also observed in cartilage repair [114].

Insights from osteoarthritis (OA) models have further illustrated cartilage healing through bone marrow mesenchymal stem cell-derived exosomes (BMSCs-EXSMs). These EVs, stimulated by TGF-β1, exhibit high levels of miR-135b, which reduces cartilage damage and pro-inflammatory factors while promoting synovial macrophage polarization to the M2 anti-inflammatory type [115]. In anterior cruciate ligament reconstruction (ACLR) models, BMSC-EXSMs expressing miR-23a-3p enhanced tendon–bone healing by reducing inflammation through targeted inhibition of the IRF1 and NF-κB pathways, demonstrating the broad repair mechanisms supported by EVs across the musculoskeletal system [116].

The potential use of EVs in doping practices has recently raised concern. Although no direct evidence is currently available, the biological properties of EVs—particularly their ability to transfer proteins, nucleic acids, and bioactive molecules—could theoretically be exploited to enhance athletic performance or accelerate recovery. Both the U.S. Anti-Doping Agency (USADA) and the World Anti-Doping Agency (WADA) have indicated that EV-based therapies could violate anti-doping regulations, particularly if they involve blood components, growth factors, or prohibited substances. Given the dynamic development of EV applications in regenerative medicine, continuous monitoring of this area is essential to prevent possible misuse in sport [117].

While current evidence remains limited, EVs offer significant potential for the development of targeted therapies in sports medicine. They may provide novel strategies for musculoskeletal repair and improved recovery following injury. The roles of EVs in musculoskeletal tissue regeneration and their potential therapeutic applications in various injury models are summarized in Table 3.

**Table 3:** The roles of EVs in musculoskeletal repair and their potential therapeutic applications in various muscle-related injuries and conditions.

Context	Role of EVs	Specific Actions and Outcomes	Reference
Muscle Injury and Regeneration	Alteration of miRNA content in EVs after exercise-induced injury	Potential marker for muscle damage and diseases like Duchenne and myotonic dystrophy	[97,100,101]
Skeletal Muscle Regeneration	EVs carrying muscle-specific miRNAs (myo-miRNAs) support muscle repair	Protection of sensitive miRNAs, involvement of satellite muscle cells (SMSCs) in repair and inflammation reduction	[98,99]
SMSC-Derived EVs	Transfer miRNAs that regulate extracellular matrix genes like MMP-9	Promotes muscle growth and supports the expression of regeneration-related proteins during tetanic contractions	[112,113]
Healing and Functional Recovery	EVs contribute to recovery post-injury	Increases muscle hypertrophy and regeneration-related proteins	[114]
Tendon and Ligament Repair	EV-based treatments modulate inflammation and support remodeling	Promotes macrophage polarization, enhances angiogenesis, and supports extracellular matrix remodeling	[114]
Cartilage Repair (OA Models)	BMSC-EXSMs promote cartilage repair by modulating inflammation and gene expression	Induces M2 macrophage polarization, reduces inflammation, upregulates miR-135b, supporting cartilage repair	[115]
Tendon-Bone Healing (ACLR Model)	BMSC-EXSMs support repair site by reducing inflammation	Enhances tendon-bone healing through inflammation reduction at the repair site	[116]
Therapeutic Potential in Sports Medicine	EVs as therapeutic agents in musculoskeletal repair	Potential for targeted therapies, especially in enhancing muscle regeneration and musculoskeletal tissue healing	[116]

## 6. Methodology Limitations in Extracellular Vesicle Studies

Enriching and characterizing EVs in exercise studies presents significant challenges due to biological and technical factors. One primary obstacle is the lack of consensus on EV terminology and standardized methods for their isolation and sample preparation, collectively known as the preanalytical phase. This phase includes key steps such as blood or sample collection techniques, centrifugation, and timing of processing, freezing, thawing, and storage. Freshly drawn blood is preferred for EV collection, as it reflects more accurate in vivo EV levels compared to frozen samples. However, plasma stored for 24 h may yield similar results to fresh samples. These methodological limitations are summarized in Table 4.

**Table 4:** Summary of recommendations and major limitations in connection to selected EV types (FCM—flow cytometry techniques; NTA—nanoparticle tracking analysis; SEM—scanning electron microscopy; TEM—transmission electron microscopy; WB—Western blotting).

Parameter	Method (Widely Used)	Recommendations	Major Limitations	Most Suitable For	References
EV Quantification (Number/Concentration)	Flow cytometry (FCM), nanoparticle tracking analysis (NTA)	Report method limit of detection; describe instrument settings; present diameter distribution.	Lack specificity for EVs; low sensitivity for small EVs.	EVs down to ~100 nm (FCM), few 100 nm (NTA).	[118,119,120]
Particle Size (Diameter)	FCM, NTA	Due to asymmetric size distribution, detailed diameter data should be presented.	Inaccurate size measurement across full EV size range.	Small EVs (~30–200 nm).	[121,122]
Morphology	SEM, TEM, cryo-TEM	Report all experimental conditions; use cryo-TEM to avoid dehydration artifacts.	Underrepresentation of larger EVs; technical limitations in conventional EM.	EVs down to ~200 nm diameter.	[118,123]
Protein Composition	Western blotting (WB), mass spectrometry	Analyze at least one marker each from categories 1, 2 (EV markers), and 3 (purity controls).	Lack of universal molecular markers for specific EV subtypes.	Applicable to all EV types.	[119,120]
Nucleic Acids	Low-input RNA sequencing, quantitative PCR (qPCR)	For qPCR, report primer/adaptor sequences; for RNA-Seq, describe RNA fragmentation and RT procedures.	No standardized methods to protect surface DNA from degradation during isolation.	Applicable to all EV types.	[122,123]

### 6.1. Blood Collection

The timing and technique of blood collection are essential in clinical and research contexts. EV levels typically increase significantly immediately after exercise but return to baseline within approximately one hour post-exercise. Standardizing blood collection is crucial; for instance, discarding the first 2–3 mL of blood and using a 21- or 22-gauge needle minimizes platelet activation. When analyzing EVs, serum and platelet-free plasma are generally more representative than whole plasma. Even commonly used anticoagulants, such as ADC and EDTA, influence EV concentration, emphasizing the need for precise documentation of blood collection methods [124].

### 6.2. Isolation and Storage

Exercise-induced increases in lipoproteins and the size overlap with particles like chylomicrons necessitate rigorous isolation methods for EVs. To date, the so-called “gold standard” has not been found; however, there are some isolation methods that seem to occur more frequently than others in the literature in the case of exercise science such as differential centrifugation/ultracentrifugation (DC/UC) and polyethylene glycol-based commercial kits (PEG; ExoQuickTM and TIER) [2]. However, research by Guo et al. (2021) suggests that size exclusion chromatography (SEC) and ultracentrifugation (UC) offer comparable purity for plasma-derived EVs, with SEC being the less time-intensive, though more costly, composition [118]. Sample storage also impacts EV integrity; long-term storage at −80 °C is recommended, but repeated thawing may activate platelets [121]. PBS with human albumin and trehalose (PBS-HAT) has been suggested to preserve samples exposed to freezing and thawing [119].

### 6.3. Characterization

After isolation, comprehensive biochemical and biophysical characterization of EVs is essential for their reliable functional analysis [122]. In 2023, the ISEV updated the MISEV2018 guidelines to promote the generation of reproducible and standardized EV research data. Although no single technique is sufficient to fully characterize EVs or assess the purity of isolates, the combination of multiple complementary methods is strongly recommended to avoid the misinterpretation of data due to the presence of co-isolated non-vesicular materials [120,123]. Recommended techniques based on the updated MISEV guidelines are summarized in Table 4.

The most widely used methods for EV characterization include Western blotting (WB), flow cytometry (FCM), various microscopy techniques (electron microscopy (EM), scanning electron microscopy (SEM), and transmission electron microscopy (TEM)), and nanoparticle tracking analysis (NTA).

WB detects the presence of canonical EV markers such as CD9, CD63, and CD81. However, it is important to note that these markers are not exclusive to specific EV subtypes (e.g., EXSMs) and may also be present on co-isolated particles [32,120].

FCM allows estimation of particle size based on light scatter and fluorescence intensity. Due to the technical limitations of conventional flow cytometers, which are optimized for cell-sized particles, small EVs (<200 nm) can be challenging to analyze. The MIFlowCyt-Ev framework [125] has introduced standardized specifications to improve reproducibility in EV FCM studies [126].

Among microscopy techniques, EM methods are adept at characterizing small EVs (sEVs), though larger EVs may be underestimated. SEM and TEM can measure individual EV size and bilayer lipid structure, while cryo-electron microscopy (cryo-TEM) addresses limitations in traditional TEM, such as sample dehydration and fixation [123,127].

NTA quantifies EV concentration and size distribution. It is effective for vesicles as small as thirty nanometers, though EVs larger than a few hundred nanometers may be challenging to quantify. However, it cannot distinguish EVs from co-isolated non-vesicular particles, making it necessary to interpret NTA results cautiously, especially in complex biofluids.

Next-generation sequencing (NGS) is increasingly used to map genetic content in EVs, including DNA, mtDNA, miRNA, and RNA, providing insights into their functional roles.

Mass spectrometry supports proteomics studies by enabling the identification and quantification of EV-associated proteins. Nevertheless, due to potential contamination by co-isolated proteins, results obtained using MS should be interpreted carefully [120].

Although the application of these techniques substantially improves the quality of EV characterization, it remains challenging to unequivocally distinguish true vesicular cargo from contaminants such as lipoproteins, protein aggregates, or other extracellular nanoparticles [25]. Therefore, we emphasize that results should be validated using multimodal approaches, combining both physical and molecular characterization strategies. A summary of recommended methods and their major limitations in relation to specific EV types is presented in Table 4.

## 7. Mechanistic and Clinical Challenges in Extracellular Vesicle Research: Barriers to Therapeutic and Diagnostic Applications

Current EV research faces substantial mechanistic limitations and challenges in translating findings into clinical applications. Mechanistically, the processes of EV biogenesis, release, uptake, and cargo selection are not fully understood, limiting the development of targeted EV-based therapies. EV subtypes have distinct functions, but their overlapping characteristics complicate selective isolation, reducing biomarker specificity [128,129].

Clinically, EVs’ scalability and reproducibility for large-scale applications are significant barriers. Isolation methods, such as ultracentrifugation and size exclusion chromatography, though widely used, are labor-intensive, costly, and yield inconsistent purity. Stability issues also arise, as EVs often have a short half-life in circulation and face rapid immune clearance, limiting therapeutic efficacy [130,131].

Additionally, non-autologous EV sources may provoke immune responses, adding to the complexity of their clinical use [132].

Regulatory challenges, such as the need for standardized production protocols, further hinder the clinical translation of EV-based treatments [129]. Without consistent quality control, gaining regulatory approval remains challenging. While EVs offer potential for diagnostics and therapeutics, overcoming these mechanistic and clinical limitations is essential for practical applications.

## 8. Extracellular Vesicles in Equine Science and Sport Horses

Interest in extracellular vesicles (EVs) in equine science is growing, but research remains limited. Methodological studies have been conducted [133,134], alongside research in equine reproduction, where EVs have been shown to reduce inflammation in endometrial cells in vitro [135,136]. Additionally, follicular fluid-derived EVs have been found to influence in vitro maturation of equine oocytes [137]. In equine metabolic syndrome (EMS), EVs have been reported to improve cell viability [138], while in laminitic ponies, EV-related changes have also been observed [139].

Recent studies highlight the pivotal role of EVs in sport horses, particularly in muscle recovery, joint health, and inflammation regulation. Mesenchymal stem cell-derived EVs (MSC-EVs) have demonstrated anti-inflammatory properties and the ability to enhance cartilage and tissue repair in vitro [140]. They contain bioactive molecules, including microRNAs like miR-21-5p and miR-451a, which contribute to tissue regeneration and immune modulation [141]. In OA, EVs from plasma and synovial fluid show altered miRNA profiles, making them potential biomarkers for joint health and injury monitoring [142]. While synovial fibroblast-derived EVs are associated with inflammation, MSC-EVs exhibit regenerative properties, making them promising therapeutic agents for OA [143]. Moreover, their anti-inflammatory and extracellular matrix-protective effects on deep digital flexor tendons (DDFTs) and navicular bone fibrocartilage (NBF) under interleukin-1β-induced inflammation in vitro have been confirmed [144]. MSC-EVs also show potential in treating suspensory ligament injuries, improving lesion filling, angiogenesis, and tissue elasticity [145].

These findings have encouraged early attempts to produce EVs for clinical applications. However, scaling up production remains a challenge. While EVs from 3D-cultured MSCs may enhance regenerative therapies, traditional 2D monolayer culture systems are impractical for large-scale manufacturing. Optimizing 3D culture methods could significantly improve BM-MSC expansion and EV yield [146].

Interestingly, EVs are now being investigated in exercise physiology. A study on Yili horses revealed that competition induces changes in blood exosome miRNA, protein, and metabolite profiles, suggesting EVs could serve as indicators of exercise-induced stress and metabolic adjustments [147]. However, studies on EV responses to physical exertion remain scarce [147,148]. De Oliveira et al. observed significant EV fluctuations in Arabian horses during a 160 km endurance race, with EV protein levels peaking two hours post-race and normalizing by 15 h, highlighting horses’ physiological responses to strenuous activity [148]. Additionally, an increased expression of eca-miR-486-5p during and after an endurance race was reported, with eca-miR-9083 expression decreasing post-race, but the study included only five horses, highlighting the need for further research.

Future research should focus on expanding these findings, as EVs could become valuable tools for monitoring training adaptation, detecting overtraining, and assessing exercise-related stress in sport horses. Collectively, these findings suggest that EVs hold significant promise as both diagnostic and therapeutic tools for managing exercise-induced injuries and optimizing equine performance.

## 9. Conclusions

EV research is enhancing personalized training, recovery, and fitness by identifying biomarkers, supporting immune response, and promoting muscle and brain health. EVs aid in tissue repair, inflammation control, and metabolic support, with stem cell-derived EVs showing potential for fast injury recovery. Addressing current challenges requires better scalability, targeting, and safety through refined isolation and delivery techniques, like ultrafiltration and engineering for tissue specificity. Future EV research will likely delve into biomarker discovery and immune modulation, enabling tailored athletic therapies with a science-driven focus on performance optimization.

## Data Availability

Not applicable.

## Abbreviations

The following abbreviations are used in this manuscript:

ACLR	Anterior cruciate ligament reconstruction
ANG1	Angiopoietin-1
ApoBDs	Apoptotic bodies
ARMMs	Arrestin domain-containing protein 1-mediated microvesicles
BMSCs-EXSMs	Bone marrow mesenchymal stem cell-derived exosomes
CAT	Catalase
DC/UC	Differential centrifugation/ultracentrifugation
DDFT	Deep digital flexor tendons
EGF	Endothelial Growth Factor
EMS	Equine metabolic syndrome
EM	Electron microscopy
EdEVs	Endothelial-derived EVs
EXSMs	Exosomes
ESCRT	Endosomal sorting complex required for transport
EVs	Extracellular vesicles
Ex-EVs	Exercise-derived EVs
FBS	Fetal bovine serum
FCM	Flow cytometry
FGF	Fibroblast growth factor
FR	Free radical
HIIT	High-intensity interval training
HLRE	High-load resistance exercise
HS	Heat shock proteins
HSF1	Heat shock factor 1
IL-3	Interleukin-3
ILVs	Intraluminal vesicles
ISEV	International Society for Extracellular Vesicles
ISS	Intraluminal shear stress
MMP-2	Matrix metalloproteinase-2
MMP-9	Matrix metalloproteinase-9
MPs	Microparticles
MSC-EVs	Mesenchymal stem cell-derived EVs
MVB	Multivesicular body
MVs	Microvesicles
Mcp1	Monocyte chemoattractant protein 1
m/lEVs	Medium/large EVs
NBF	Navicular bone fibrocartilage
NGS	Next-generation sequencing
NTA	Nanoparticle tracking analysis
OA	Osteoarthritis
OTS	Overtraining syndrome
PBS-HAT	PBS with human albumin and trehalose
PDGF	Platelet-derived growth factor
PEVs	Platelet-derived EVs
PGC-1a	Peroxisome proliferator-activated receptor gamma co-activator 1-alpha
ROS	Reactive oxygen species
SEM	Scanning Electron Microscopy
sEVs	Small EVs
SIRT1	Sirtuin-1
SM	Skeletal muscle
SM-EVs	Skeletal muscle-derived EVs
SMSCs	Satellite muscle cells
SOD2	Superoxide dismutase 2
TEM	Transmission electron microscopy
USADA	USA. Anti-Doping Agency
VEGF	Vascular endothelial growth factor
WB	Western blotting
WADA	World Anti-Doping Agency
